# Application of cell-derived vesicle-based biomimetic drug delivery system in tumor therapy

**DOI:** 10.3389/fphar.2025.1632361

**Published:** 2025-07-11

**Authors:** Yan Mei, Maochang Liu, Meng Mei

**Affiliations:** Department of Pharmacy, Wuhan Children’s Hospital (Wuhan Maternal and Child Healthcare Hospital), Tongji Medical College, Huazhong University of Science and Technology, Wuhan, China

**Keywords:** cell-derived vesicles, drug delivery, tumor therapy, hybrid nanovesicles, biomimicry

## Abstract

Cell-derived vesicles play a crucial role in intercellular information transmission and serve as vital carriers facilitating cell-to-cell communication. These vesicles contain specific biological information from their parental cells, enabling them to transport proteins, cytokines, mRNA, and DNA to other cells or organs. Presently, cell-derived vesicles have extensive application in inhibiting tumor growth and metastasis, as well as in tumor diagnosis and early screening. Numerous studies have highlighted the potential of cell-derived vesicles as drug delivery systems, showcasing advantages such as enhanced biocompatibility, natural active targeting, improved systemic circulation, and greater efficacy compared to conventional drug delivery carriers. In this article, we aim to explore the utilization of cell-derived vesicles as drug delivery systems in tumor therapy, focusing on various drug-loading techniques and analyzing the advantages and characteristics of different types of cell-derived vesicles. Our findings will provide valuable assistance in the development of drug delivery systems based on these vesicles.

## 1 Introduction

Since the first discovery of extracellular vesicles in the 1940s, research on cell-derived vesicles has gradually become hot, especially in the 21st century. Due to the discovery of the unique biological characteristics of extracellular vesicles, there was an explosive increase in research on the treatment and detection of various diseases using cell-derived vesicles (Zhang et al., 2023). Because of the discovery of the extracellular vesicle transport regulation mechanism in cells, James E. Rothman and three others shared the Nobel Prize in Physiology and Medicine in 2013. In general, cell-derived vesicles can be classified into exosomes, macrovesicles and apoptotic bodies, which based on the vesicle diameter and size (György et al., 2011; Shao et al., 2018; Massa et al., 2020; Buzas, 2023). Studies have confirmed that cell-derived vesicles are important information carriers for intercellular communication (Robbins and Morelli, 2014; Becker et al., 2016). Cell-derived vesicles can transmit biological information such as protein and nucleic acid in different cells, and they are involved in pathological processes such as cancer recurrence and metastasis, angiogenesis and repair, brain injury and stroke, infectious diseases, and so on (Todorova et al., 2017; Karpman et al., 2017). Cell-derived vesicles possess membrane structures and protein information similar to that of parental cells, endowing them with the ability to actively target specific cells and tissues. This is known as the homing effect of cell-derived vesicles. For example, when tumor cell-derived vesicles are distributed within the body, they can specifically target the same type of tumor tissue, resulting in a significantly higher distribution in tumor than in other tissues or organs (El An et al., 2013; Maacha et al., 2019). In addition, cell-derived vesicles have high biocompatibility, long systemic circulation time, and a closed structure formed by natural phospholipid bilayers, which have great potential in drug delivery (Tarasov et al., 2021; Antimisiaris et al., 2018). At present, there have been many studies on the use of extracellular vesicles to carry various drugs for the treatment of cancer, cardiovascular disease, diabetes and brain disease, among which the use of extracellular vesicles to carry drugs for cancer treatment is the most host (Tai et al., 2018; Boulanger et al., 2017; Ramos-Zaldívar et al., 2022).

Chemotherapy, radiotherapy, surgery and other traditional cancer treatment methods can achieve effective treatment of some tumors, but for malignant tumors such as malignant melanoma, pancreatic cancer, triple-negative breast cancer, the treatment effect is poor and side effects are serious (Wu et al., 2022; Sharma et al., 2016; Li et al., 2022). In recent years, with the gradual deepening of research on the tumor immune microenvironment, regulating the activity of immune cells activating anti-tumor immune responses, and stimulating immune cells to kill tumor cells have gradually become the mainstream research direction of tumor treatment, and have given rise to tumor immunotherapy (Vader et al., 2014). Cell-derived vesicles can carry parental cells functional molecules (such as tumor cell antigens, immune cell inducible factors) and deeply participate in the regulation of anti-tumor immune responses. In addition, it is equipped with chemotherapy drugs, photodynamic therapy drugs to achieve immune combination therapy, greatly enhancing the inhibitory effect on malignant tumors (Tarasov et al., 2021; Qiu et al., 2018). It is expected to provide guidance for clinical cancer treatment by developing drug delivery systems with different cell-derived vesicles for immunotherapy of malignant tumors.

Here, this review summarizes the research status of drug delivery systems based on extracellular vesicles in cancer treatment in recent years. It provides a detailed introduction to the research progress of single cell-derived vesicle and hybrid vesicle drug delivery systems, and introduces the drug loading methods of vesicles. In addition, to further analyze the clinical translational potential of various vesicle drug delivery systems, which might provide guidance for the subsequent development of clinically translatable cell derived vesicle biomimetic drug delivery systems.

## 2 Single cell derived vesicle drug delivery system

### 2.1 Tumor cells

**FIGURE 1:**
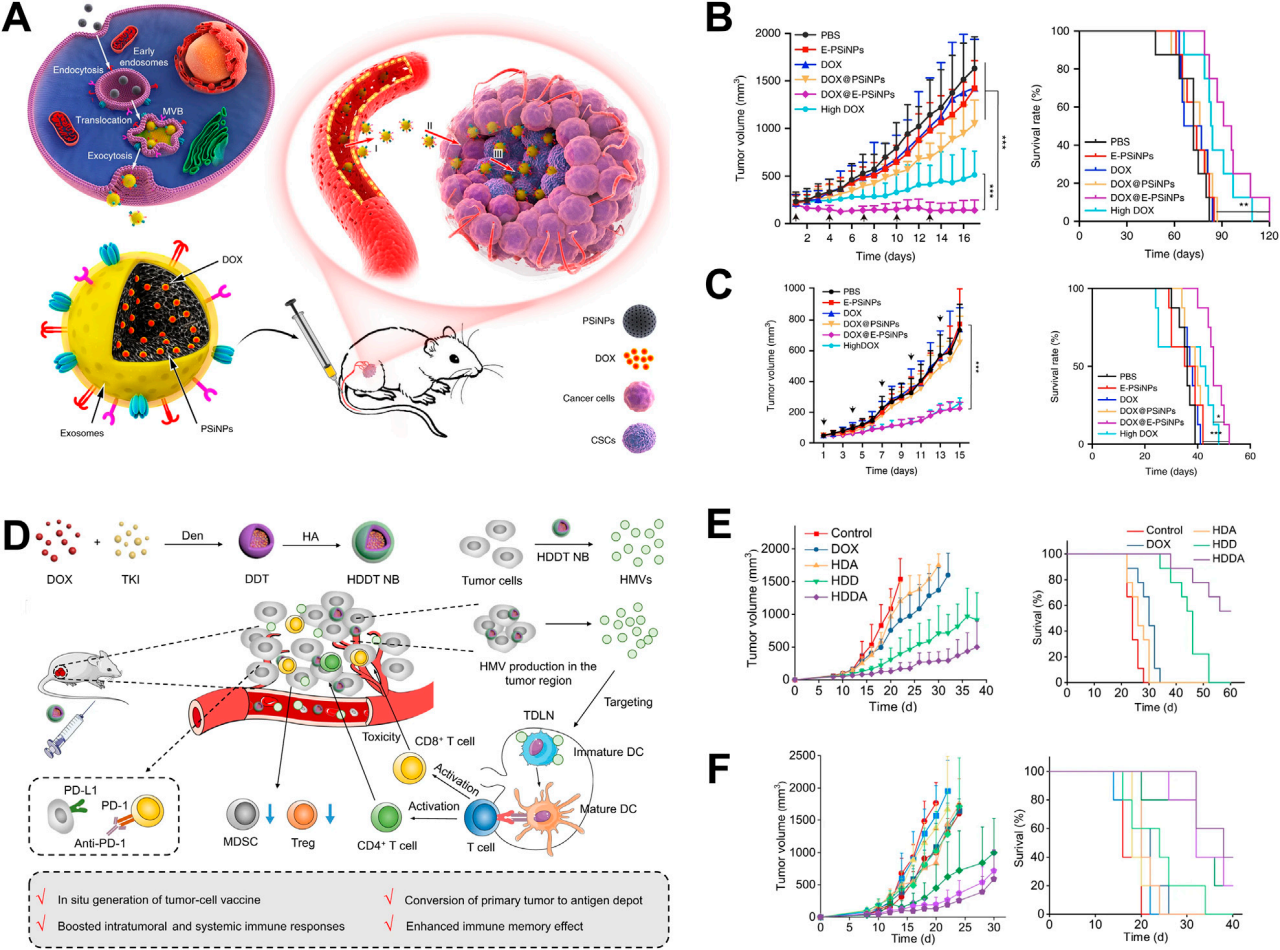
Application of tumor cell-derived vesicles as drug delivery systems in tumor therapy. **(A)** The application of tumor cell-derived vesicles loaded with Dox in tumor treatment (Yong et al., 2019); **(B)** Drug-loaded vesicles inhibited 4T1 tumor growth and prolong survival time (Yong et al., 2019); **(C)** Drug-loaded vesicles inhibited H22 tumor growth and prolong survival time (Yong et al., 2019); **(D)** The application of vesicular vaccines generated *in-situ* in tumor treatment (Guo et al., 2022); **(E)** The vesicular vaccine inhibits the growth of 4T1 tumors and prolongs survival time (Guo et al., 2022); **(F)** Drug-loaded vesicles inhibited the growth of B16F10 tumors and prolong survival time (Guo et al., 2022).

Tumor cell-derived vesicles carry parental cell biological information and have active targeting characteristics towards the same type of tumor tissue, promoting the enrichment of drugs in tumor tissue, enhancing therapeutic efficacy and reducing toxicity (Liu et al., 2021). Yong constructed a tumor cell-derived extracellular vesicle, which encapsulated porous silicon biomimetic nanoparticle and were used to carry the chemotherapy drug doxorubicin (Dox) (Yong et al., 2019). The biomimetic nanoparticle utilized the homologous targeting properties of tumor cell-derived extracellular vesicles to specifically target tumor cells and tumor stem cells, release Dox, and kill tumor cells. In addition, the adhesion molecule CD54 on the surface of the exosome promotes the drug-loaded nanoparticles to deeply penetrate the tumor tissue, and effectively inhibited the growth of subcutaneous tumors of liver cancer, breast cancer and melanoma (Figures 1A–C). Wang constructed a biomimetic drug delivery system (Bi2Se3/Dox@MPs) formed with tumor cell-derived vesicles, Bi2Se3 nanodots and chemotherapy drug Dox (Wang et al., 2020). This biomimetic drug delivery system could achieve enrichment and deep infiltration of tumor tissues after biodistribution. In addition, Bi2Se3 carried by Bi2Se3/Dox@MPs possessed the photothermal effect, and could effectively inhibit the growth of H22 liver cancer with the combined treatment of Dox. Furthermore, Qin used the redox environment of tumor cells to reduce HAuCl4 and generate gold nanoparticles, which were then expelled from the tumor cell membrane to form gold nanoparticles (Au@MC38) (Qin et al., 2021). Due to the homing characteristics of tumor cell-derived vesicles, Au@MC38 could achieve active targeting of tumor tissue, promoted the enrichment of gold nanoparticles in tumor tissue, and combined with photothermal therapy to induce immunogenic cell death (ICD) of tumor cells, which generating *in-situ* cancer vaccine effect, thereby activating cellular immune response and effectively inhibiting tumor growth.

Tumor cell-derived vesicles carry tumor antigen and other bio-information, which could induce APC maturation and present tumor antigen information to T cells, activating cytotoxicity T lymphocytes (CTLs) and further killing tumor cells (Zheng et al., 2023). Guo used polymer nanoparticles loaded with Dox and tyrosine kinase inhibitors to stimulate tumor cells to produce a large number of vesicles (HMVs) in tumor bearing mice (Guo et al., 2022). HMVs carried tumor antigens and exhibited *in-situ* tumor vaccine effect, which could activate the cellular immune response in mice, effectively inhibited tumor cell growth, and extended the survival time of tumor bearing mice (Figures 1D–F). In addition, Yang prepared an effective tumor vaccine that activated anti-tumor immune response by encapsulating tumor cell membranes with polymer nanoparticles loaded with immune adjuvant R848 (NP-R@M-M) (Yang et al., 2018). After being ingested by dendritic cells (DCs), the tumor antigens carried by the nanoparticles are processed by DCs and the antigenic determinants were expressed on DCs. At the same time, the immune adjuvant R848 carried in the nanoparticles activated DCs, enhances the antigen presentation ability, and better activated CTL in lymph nodes. The activated CTLs further infiltrates into tumor tissue, killing tumor cells and inhibited tumor growth. In addition, Jin has conducted clinical trials on tumor cell-derived vesicles in the field of malignant treatment and achieved significant results. The team’s pioneering “drug loaded vesicle therapy for tumors” has demonstrated good safety and effectiveness in the treatment of malignant pleural effusion (Guo et al., 2019).

In addition, tumor cell-derived vesicles inhibit the function of immune cells through various mechanisms, such as carrying FasL and TRAIL molecules, directly inducing apoptosis of dendritic cells (DCs), effector CD4^+^ and CD8^+^ T lymphocytes, or inducing regulatory T cell expansion by carrying upregulated circular RNA GSE1, thereby suppressing anti-tumor immune responses (Markov et al., 2019). Furthermore, tumor derived vesicles could Block the differentiation of myeloid progenitor cells into CD11c + DCs and directing them towards differentiation into myeloid derived suppressor cells, or inhibiting the maturation and migration of DCs, leads to immune tolerance and facilitates tumor immune escape (Peng et al., 2011).

### 2.2 Erythrocyte

There was large number of erythrocytes in the systemic circulation, and possess the characteristics of none nucleus and high plasticity, making them an excellent choice for biomimetic drug delivery materials. Zhang first proposed the use of red blood cell membrane coated nanoparticles for drug delivery research in 2011 (Hu et al., 2011). With the development of more than decades, research on erythrocytes-derived vesicles has emerged one after another. Researchers have developed a variety of drug delivery systems based on erythrocytes-derived vesicles. Wang developed a biomimetic drug delivery system containing small interfering RNA (siRNA), Dox, and aptamers using the long circulation characteristics of erythrocytes-derived vesicles (Wang et al., 2019). Adaptor modification endows vesicles with active targeting, promoting their enrichment in tumor tissue and increasing drug content within the tumor tissue. Meanwhile, for multidrug-resistant tumors, the siRNA corresponding to P-glycoprotein (P-gp) can effectively reduce the expression of P-gp on tumor cells, reduce their resistance to Dox, enhance their tumor killing ability, and thus improve the effectiveness of tumor treatment. In addition, Guo used erythrocytes membrane to encapsulate PLGA nanoparticles, enhancing their long-term circulation properties, and modified them with small molecules of mannose, endowing them with the ability to actively target DC (Guo et al., 2015). In addition, with loading on tumor antigens and immune adjuvant MPLA, this delivery system can further promote DC maturation and tumor antigen presentation, thereby activating CTL and effectively killing tumor cells.

### 2.3 Macrophages

Macrophages, as important immune cells in the immune system, are deeply involved in anti-tumor immune responses (Mantovani et al., 2022). According to differences in biological functions, macrophages are often divided into two subtypes, M1 and M2. M1 macrophages have the ability to promote inflammatory response. In the tumor immune microenvironment, M1 macrophages can promote the level of inflammation in tumor tissue and enhance the immune activity. In addition, M1 macrophages also have antigen presentation ability, and present tumor antigens to follicular helper T cells and further activate B cells, then, activate anti-tumor humoral immune response (Germic et al., 2019). M2 macrophages, as an inflammatory suppressive cell, which play an important role in wound healing, tissue repair, and the construction of immunosuppressive tumor microenvironment (Louiselle et al., 2021). In tumor immunotherapy, it is often achieved by educating the phenotype of M2 macrophages within tumor tissue or directly killing M2 macrophages to reduce the immunosuppressive effect of the tumor microenvironment and enhance the killing ability of other immune cells against tumor cells (Kumari and Choi, 2022; Xia et al., 2020).

**FIGURE 2:**
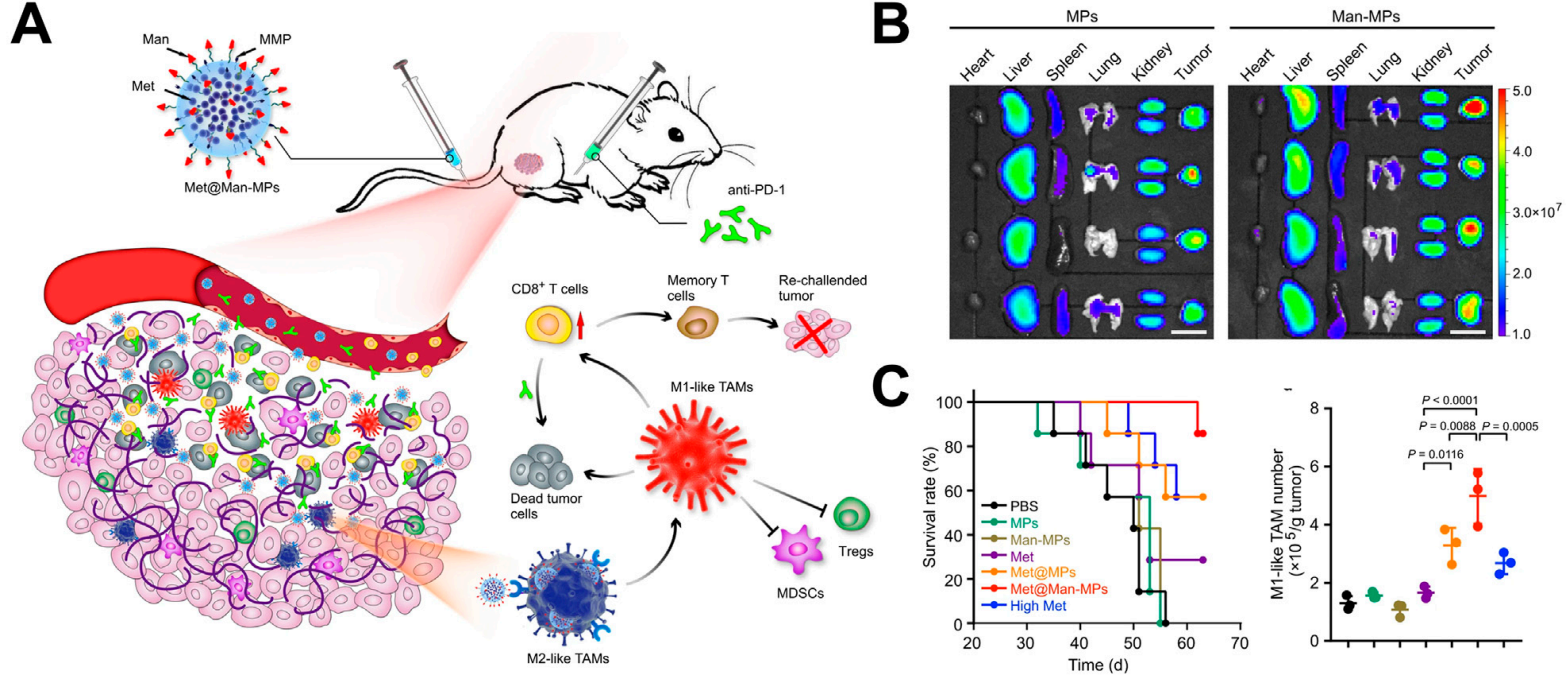
Application of macrophage-derived vesicles as drug delivery systems in tumor treatment (Wei et al., 2021). **(A)** The application of metformin-loaded macrophages-derived vesicles in tumor treatment; **(B)** Drug-loaded vesicles possessed tumor tissue targeting property; **(C)** Drug-loaded vesicles upregulated the proportion of M1 macrophages in tumor tissue and prolonged survival time.

Choo constructed an M1 type macrophage extrusion vesicle that can carry pro-inflammatory mRNA and proteins (Choo et al., 2018). These vesicles could polarize M2 macrophages to M1 macrophages in tumor tissue, which alleviated the inhibitory immune state within the tumor microenvironment, and effectively inhibit tumor growth. In song kinds of tumors, M1 macrophage-derived vesicles showed the insufficient ability to regulate the phenotype of M2 tumor associated macrophages. Therefore, M1 macrophage-derived vesicles are used to carry drugs related to macrophage polarization, further enhanced their ability to regulate macrophage phenotype. Wei prepared M1 macrophage-derived vesicles loaded with metformin by UV irradiation and constructed a biomimetic drug delivery system (Wei et al., 2021). These vesicles could target M2 macrophages with mannose modification (Met@Man-MPs) (Figure 2A). In the bio-distribution experiment, Met@Man-MPs demonstrated good tumor targeting ability (Figure 2B). Through immunological analysis, it was found that Met@Man-MPs could effectively regulate the phenotype of M2 macrophages, recruit CD8^+^ T cells, and prevent suppressive immune cells (myeloid suppressor cells and regulatory T cells) from entering tumor tissue (Figure 2C). This treatment strategy could enhance the immune activity of tumor microenvironment and promote killing tumor cells by immune cells. In addition, in order to enhance the antigen presentation function of M1 macrophages, Wang constructed a tumor cell macrophage chimeric exosome (Wang et al., 2021). After macrophages swallowed the nucleus of tumor cells, tumor antigen-related genes utilized the protein synthesis function within macrophages to synthesize tumor-related antigens, which further expressed on the surface of macrophages. Finally, M1 macrophages-derived exosomes with presenting tumor antigens were. The chimeric antigen exosomes effectively targeted tumor tissue and draining lymph nodes, regulated the tumor immunosuppressive microenvironment, and activates anti-tumor immune responses, which effectively inhibiting tumor growth and metastasis.

### 2.4 Dendritic cells

DCs possess the strongest antigen presentation ability among APCs, therefore, DC-derived vesicles are often used to present antigens and activate anti-tumor immune responses (Bommareddy et al., 2018). Wu used tumor cell-derived vesicles to shock DCs, induced them to express tumor antigen determinants on the surface of DCs membranes (Wu et al., 2017). They further co-incubated Dox with activated DCs and generated DC-derived vesicles loaded with Dox through UV irradiation. After subcutaneous distribution, the DC-derived vesicles are highly enriched in tumor tissue. In addition, Dox was successfully carried to tumor tissue to kill tumor cells and produced ICD effects. DC-derived vesicles could also directly stimulate DC maturation and activate cellular immune responses. This treatment strategy could effectively inhibit the growth of melanoma and the occurrence of lung metastasis. Zhang also constructed a DC-derived vesicle loading system (Zhang et al., 2019). Firstly, HAuCl4 solution was co-incubated with melanoma cells, and gold ions were reduced to gold nanoparticles using reducing substances within the tumor cells. The gold nanoparticles coated with the tumor cell membrane were collected by centrifugation, and further stimulated DC maturation to obtain gold nanoparticles coated with the matured DC cell membrane (AuNP@DC_B16F10_). AuNP@DC_B16F10_ could lead the targeting of tumor tissue and draining lymph nodes, which promoted DC maturation, and activated anti-tumor immune responses. In addition, after accumulating in tumor tissue, gold nanoparticles generate a photothermal effect through near-infrared light irradiation, and finally killing tumor cells.

To improve the immune activation efficiency of DC derived vesicles, Liu constructed DC-derived vesicles that simultaneously expressed tumor antigens and major histocompatibility class I complexes, B7 co-stimulatory molecules, and anti PD1 antibodies through lentiviral transfection (Liu et al., 2022). Through multiple DCs co-stimulatory signal transmission, systematic activation of anti-tumor immune responses was achieved. This DC-derived vesicle could directly activate initial T cells, omitting the step of DC transmitting antigens *in vivo*, greatly improved the efficiency of immune activation. In addition, it could also reactivate the depleted T cells with anti-PD1 antibodies. This immune activation strategy could directly activate the immune responses and reverse immune tolerance, providing new ideas for the development of clinical DC-derived vesicular vaccines.

In addition, in some cases, dendritic cell-derived vesicles may carry self-antigens. When these vesicles are recognized by self-reactive T cells, they may trigger autoimmune reactions and attack normal tissue cells (Mbongue et al., 2014). Research has found that extracellular vesicles derived from dendritic cells can induce the expansion of regulatory T cells, thereby inhibiting the function of effector T cells, leading to immune tolerance, and affecting anti-tumor immune response (Whiteside, 2016; Wu et al., 2020).

## 3 Hybrid vesicle drug delivery system

With further research on the single cell-derived vesicular drug delivery systems, it has gradually been found that the biological functions of single cell-derived vesicles are insufficient to regulate the tumor immune microenvironment in multiple dimensions. In addition, vesicular drug delivery systems need more stringent requirements for the properties of the drugs compared to traditional drug delivery carriers, like liposomes. Therefore, the research of hybrid vesicles system is gradually emerging to broaden the applicability of vesicle drug delivery systems.

### 3.1 The hybridization of vesicles

**FIGURE 3:**
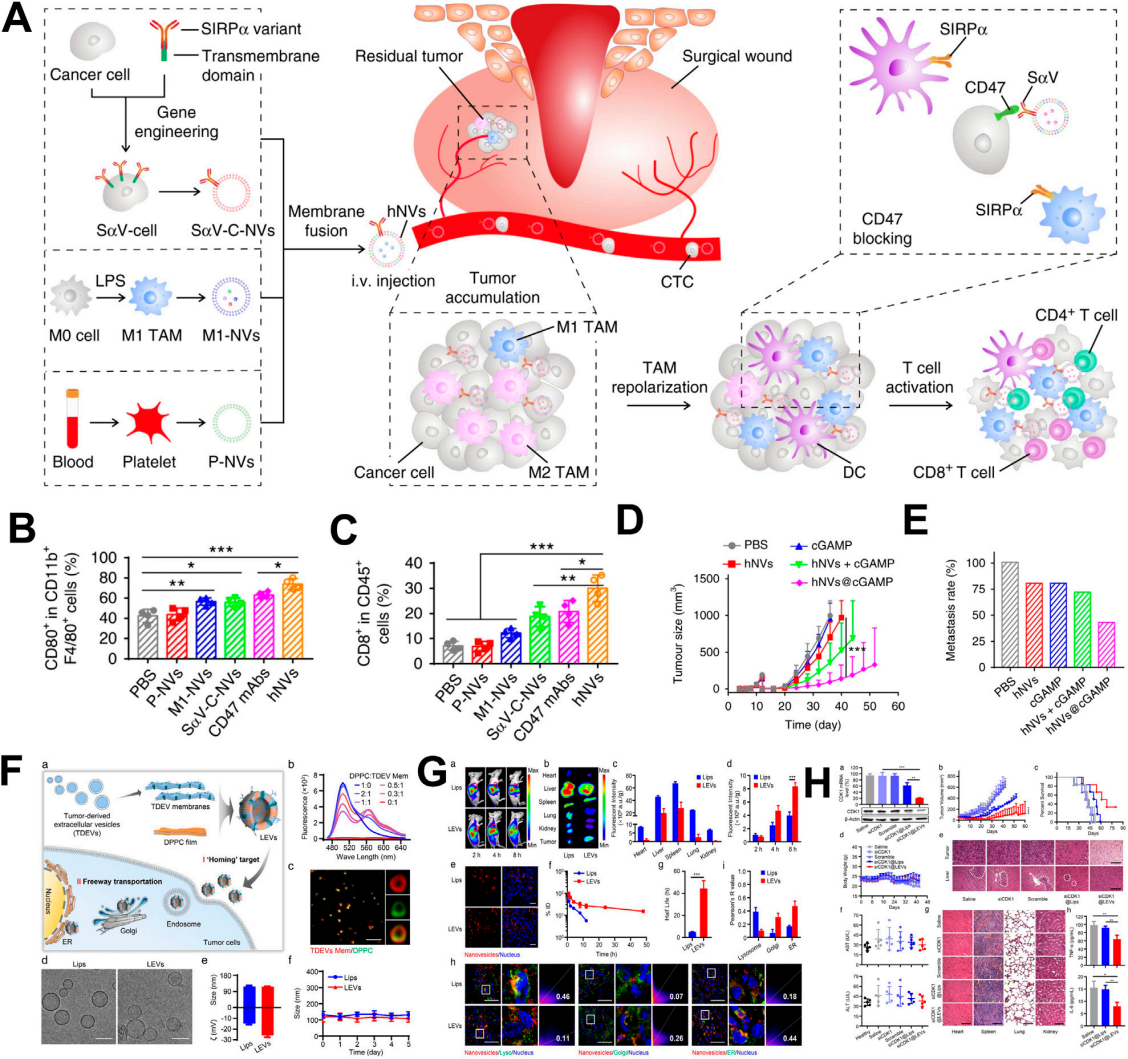
The application of hybrid vesicles as drug delivery system in tumor treatment. **(A)** The application of hybrid vesicles based on the fusion of macrophage vesicles, tumor cell membrane vesicles and platelet derived vesicles in tumor therapy; **(B)** Hybrid vesicles promoted polarization of tumor associated macrophages towards M1 macrophages; **(C)** Hybrid drug-loaded vesicles upregulated the proportion of CD8+ T cells; **(D)** Hybrid vesicles inhibited tumor growth and prolong survival time; **(E)** Hybrid vesicles inhibited tumor metastasis; **(F)** The application of hybrid vesicles based on the fusion of tumor cell membrane vesicles and liposomes were used in tumor therapy; **(G)** Hybrid vesicles had tumor targeting properties; **(H)** Hybrid vesicles inhibited tumor growth and prolong survival time. **(A–E)** reprinted from Rao et al. (2020); **(F–H)** reprinted from Zhou et al. (2022).

To change the singularity of the biological function of the vesicular biomimetic drug delivery system, two or more kinds of cell-derived vesicles are hybrid. The hybrid vesicles possess multiple biological functions, and improved their drug delivery and immune regulatory performance. Han developed a hybrid vesicle system based on the fusion of tumor cell membrane vesicles and erythrocytes membrane vesicles (Han et al., 2019). These hybrid vesicles possessed the long circulation characteristic of erythrocytes and the ability of tumor cell to carry tumor antigens. In the *in vivo* distribution experiment, hybrid vesicles exhibited a higher distribution ability in the spleen. Further immune analysis revealed that hybrid vesicles could specifically activate NK cells, CD4^+^ T cells, CD8^+^ T cells, and CD19^+^ B cells, indicating their multiple immune activation abilities. Tumor inhibition experiments had shown that hybrid vesicles could significantly inhibit tumor growth and prolong the survival time of tumor-bearing mice after combined with αPD-L1. Research had shown that M1 macrophage-derived vesicles could regulate the immunosuppressive microenvironment of tumor tissue and platelet derived vesicles could actively target traumatic tissue (Wang et al., 2017). Rao constructed hybrid vesicles based on tumor cell membrane vesicles, M1 macrophage-derived vesicles and platelet derived vesicles, and modified SIRPα protein on the hybrid vesicles through genetic engineering to block the immune escape pathway of tumor cells (Figure 3A) (Rao et al., 2020). *In vivo* distribution experiments showed that hybrid vesicles possessed tumor resection site targeting properties. Further immune analysis revealed that hybrid vesicles could induce the repolarization of M2 macrophages towards M1 type in the tumor microenvironment (Figure 3B). These vesicles could activate CD4^+^ and CD8^+^ T cells (Figure 3C), alleviate the immunosuppressive effect of the tumor microenvironment and enhance the intensity of anti-tumor immune responses. In addition, tumor inhibition experiment showed that hybrid vesicles could effectively inhibit the recurrence and metastasis of melanoma and triple-negative breast cancer after surgery (Figures 3D,E).

The hybrid cells strategy was another method to prepare hybrid vesicles. Liu constructed a fusion cell between tumor cells and DCs using hybridoma technology, and used this fusion cell membrane to construct hybrid vesicles for coating polymer nanoparticles (Liu et al., 2019). These hybrid vesicles could directly activate CD8^+^ T cells or activate DCs, and then activated CD8^+^ T cells by active DCs. This strategy could achieve multi-channel activation of the immune system and effectively inhibiting tumor growth. These researches provided new ideas for the preparation of hybrid vesicles from different cell sources, and showed high value.

### 3.2 The hybridization between vesicles and liposomes

Liposomes, as traditional drug delivery carriers, have a wide range of applications in the delivery of hydrophilic and hydrophobic small molecule drugs, as well as large molecule drugs such as nucleic acids and proteins. The listed liposome drug products include doxorubicin liposome (Bavli et al., 2019), daunorubicin liposome (Eckardt et al., 1994), bupivacaine liposome (McAlvin et al., 2014), irinotecan liposome (Tran et al., 2017) and COVID-19 liposome vaccine (BNT162b2) (Lamb, 2021). Cell-derived vesicles have a natural phospholipid bilayer structure and a closed chamber, similar to the structure of liposomes. However, due to their origin from natural cells, most drugs could only be loaded through post loading, which greatly limited the further development of cell-derived vesicles in drug delivery (Herrmann et al., 2021). Compared to cell-derived vesicles, liposome-based drug delivery methods were diverse and mature, but they did not possess the characteristic of carrying multiple biological information. Therefore, combining the advantages of liposome drug delivery with the rich biological information carried by cell-derived vesicles, developing a hybrid drug delivery system between liposomes and cell-derived vesicles will broaden the development of cell-derived vesicles in drug delivery.

Hu used liposomes to carry Dox and fused it with tumor cell-derived vesicles to construct a biomimetic drug delivery system (Hu et al., 2021). These hybrid vesicles delivered tumor antigen information to the DC, induces DC maturation, and activated anti-tumor immune responses. Meanwhile, the hybrid vesicles also had tumor tissue targeting properties, which could specifically deliver Dox to tumor tissue, and induced ICD effects and further activate cellular immune responses. In addition, when used in combination with the immune checkpoint inhibitor αPD-1, hybrid vesicles could significantly inhibit tumor cell growth. In the delivery of nucleic acid drugs, tumor cell-derived vesicles showed unique advantages. Their homologous targeting ability enabled targeted delivery of nucleic acid drugs to tumor tissue. However, tumor cell-derived vesicles carrying nucleic acid drugs required the use of electroporation, and their inherent potential carcinogenic risk seriously hinders their delivery of nucleic acid drugs for cancer treatment. Zhou extracted the cell membrane of tumor cell-derived vesicles and fused them with DPPC liposomes to construct hybrid vesicles carrying siRNA (Figure 3F) (Zhou et al., 2022). This hybrid vesicle possessed the homologous targeting ability of tumor cell-derived vesicles and avoids potential carcinogenic risks (Figure 3G). In addition, DPPC liposomes could simply and efficiently carry siRNA. Compared to traditional liposomes, hybrid vesicles could bypass the endosome degradation process and directly transmit siRNA through the Golgi apparatus and endoplasmic reticulum pathways, effectively inhibiting tumor growth and prolonging survival (Figure 3H). Related research has opened innovative ideas for the efficient delivery of nucleic acid drugs.

Co-extrusion could reduce the size and distribute the vesicles more evenly, improving their uniformity and stability. By using a micro-push machine and a polycarbonate membrane with a specific aperture to compress cell membrane vesicles, smaller and more uniform vesicles can be obtained. And the surface proteins of the compressed vesicles may undergo partial loss or conformational changes, but they could still maintain a certain level of biological activity and targeting ability (Jo et al., 2014). Ultrasonic treatment can make the size distribution of vesicles uniform, but it may cause certain damage to the structure of vesicles, making them prone to aggregation during storage and reducing stability. But ultrasound can improve the loading efficiency of drugs, enhance the cellular uptake ability of vesicles, thereby improving the efficiency of drug delivery and therapeutic effects (Haney et al., 2015; Ki et al., 2016). Chemical inducers can increase the fluidity of cell membranes and promote cell fusion, but some chemical inducers may cause certain damage to the membrane structure of vesicles, affecting their stability (Danilushkina et al., 2023).

### 3.3 Others

The latest research showed that most solid tumor tissues contain a trace of bacteria, among which some special bacteria have high biocompatibility and immunogenicity, which could induce immune cell infiltration into tumor tissue and produce strong tumor inhibitory effects (Nejman et al., 2020; Fu et al., 2022; Goto et al., 2023). Chen developed a hybrid vesicle based on the fusion of E. coli bacterial outer membrane and tumor cell membrane vesicles (Chen et al., 2021). The bacterial outer membrane had a natural adjuvant effect, transmitting danger signal molecules to the body, thereby stimulated DCs, enhanced its uptake and presentation of tumor antigens, and strongly activated anti-tumor specific immune responses. In the postoperative tumor resection model, this hybrid vesicle effectively activated tumor specific immune response and inhibited tumor recurrence. Related research had the potential to be applied in various relapsed solid tumors, providing effective assistance for the development of personalized tumor treatment plans in clinical practice.

In addition, the advantages, disadvantages and applicable scenarios of different types of vesicles were summarized in Table 1.

**TABLE 1:** The summary of the advantages, disadvantages and applicable scenarios of different types of vesicles.

Types	Advantages	Disadvantages	Applications
Tumor cell derived vesicles	Tumor target, Tumor bio-information, Natural sources	Potential carcinogenicity	Tumor immunotherapy, Drug delivery
Erythrocyte derived vesicles	No content, Natural sources, Readily available	Poor editing quality	Drug delivery with long circulation time
Macrophages derived vesicles	Natural sources, Immunoregulation, Macrophages polarization, Humoral immunity activation	Not easily obtainable, High cost	Tumor immunotherapy, Microenvironment regulation, Drug delivery
DC derived vesicles	Natural sources, Immunoregulation, T cells activation, Cellular immunity activation	Not easily obtainable, High cost	Tumor immunotherapy, Drug delivery
Hybridization of vesicles	Natural sources, Functional integration	Complex preparation, High cost	Disease treatment, Immune activation, Drug delivery
Hybridization between vesicles and liposomes	Functional integration, Hydrophobic drug carrier, Strong modifiability, Readily available	Complex preparation, Organic residue	Drug delivery, Disease treatment, immunoregulation

## 4 Drug loading methods for vesicular drug delivery systems

The drug loading methods of cell-derived vesicle could be mainly divided into pre-processing methods and post-processing methods, which was based on the different processing orders (Wang et al., 2022).

### 4.1 Pre-processing method

Pre-processing method refers to co-culturing drugs with cells or expressing target drugs (proteins, nucleic acids and so on) into cells through gene transfection before vesicle separation, followed by preparing drug-loaded vesicles through extrusion or starvation methods.

#### 4.1.1 Co-incubation method

Co-incubation method refers to the cultivation of drugs with cells, and inducing the secretion of drug encapsulated vesicles by extrusion, starvation or UV irradiation after sufficient drug uptake by cells. Wei co-incubated polyethylene glycol lipids coupled with mannose and metformin with M1 macrophages, and these macrophages-derived vesicles carrying small molecules of mannose and metformin (Kumari and Choi, 2022). Qin co-incubated cells with HAuCl4, using intracellular reducing substances to reduce Au3^+^ to gold atoms and aggregate to form gold nanoparticles (Qin et al., 2021). The gold nanoparticles were then carried by vesicles through extracellular efflux. In addition, cell-derived vesicles carrying chemotherapy drugs can also be isolated by co-culturing paclitaxel (PTX) and Dox with cells (Pascucci et al., 2014; Toffoli et al., 2015). This method is simple and convenient, but the loading efficiency is relatively low, usually less than 10%, and may affect the composition of EVs (Han et al., 2021).

#### 4.1.2 Transfection method

Cell-derived vesicles can be used to carry proteins or nucleic acid drugs, and transfection is used to express the target protein or nucleic acid in cells. Li induced the expression of microRNA-424 (miR-424) in mesenchymal stem cells (MSCs) through transfection, and collected vesicles carried miR-424 to inhibit tumor neovascularization, thereby inhibiting tumor growth and metastasis (Li et al., 2021). Yu overexpressed TIGIT protein on the surface of HEK293T cells through transfection and constructed HEK293T cell and platelet-derived hybrid vesicles (Yu et al., 2022). The TIGIT protein on the surface of the hybrid vesicles could inhibit the immunosuppressive effect of CD154 protein on CD8^+^ T cells on necrotic tumor cells. This method can load large molecular substances with relatively high efficiency, but requires transfection reagents, and the efficiency is affected by transfection efficiency (Han et al., 2021).

### 4.2 Post-processing method

Post-processing method refers to first separating the vesicles, and then loading drugs into the vesicles through co-incubation, ultrasound, electroporation, freeze-thaw methods and so on.

#### 4.2.1 Co-incubation method

Cell-derived vesicles were collected and incubated with high concentration drugs, then centrifuged to collect drug-loaded vesicles. This method is suitable for small molecule drugs. Kuerban prepared Dox-loaded vesicles by co-incubating Dox and bacterial-derived vesicles (Yu et al., 2022). These vesicles could regulate macrophage phenotype and effectively inhibited the growth of non-small cell lung cancer. Zhu collected embryonic stem cell-derived vesicles and incubated them with polyethylene glycol lipid conjugated targeting peptides (cRGD) and PTX to prepare cRGD modified paclitaxel loaded vesicles (Zhu et al., 2019). These drug-loaded vesicles could achieve tumor targeting and effectively inhibit tumor growth. Co-incubation method is simple to operate, but the drug loading efficiency is relatively low, which usually less than 10% (Gaurav et al., 2021).

#### 4.2.2 Ultrasonic method

Ultrasonic method refers to the use of ultrasound to destroy the structure of vesicles, and encapsulate drugs into the vesicles during the process of vesicle restructuring, and achieve drug loading inside the vesicles. Yu prepared hybrid vesicles loaded with oxaliplatin by ultrasound method, which efficiently loaded oxaliplatin and effectively suppressed postoperative tumor recurrence (Yu et al., 2022). In addition, Haney also constructed macrophages derived vesicles loaded with chemotherapy drugs Dox or PTX using ultrasound (Haney et al., 2020). This method is easy to operate and has a relatively high drug loading capacity. This method can improve loading efficiency, usually up to about 30%, but may damage the structure and function of vesicles, resulting in low efficiency for some hydrophobic drugs (Gaurav et al., 2021).

#### 4.2.3 Electroporation method

Electroporation method refers to the use of pulsed current to drill holes on the vesicle membrane under the action of an external electric field, allowing small molecule drugs, nucleic acid drugs, and proteins to enter the vesicle (Tian et al., 2014; Hadla et al., 2016). Electroporation is the preferred method for loading nucleic acid drugs into vesicles. Zhang loaded miRNA-155 antisense oligonucleotides into erythrocytes-derived vesicles using electroporation and used them for the treatment of acute liver failure (Zhang et al., 2020). Animal experiments had shown that erythrocytes-derived vesicles can deliver miRNA-155 to liver macrophages and effectively alleviate macrophage dependent acute liver failure. This method can load large molecular substances, but it will damage the integrity of the vesicle structure, causing some vesicle contents to leak, and the operation is complex and costly. The loading efficiency can reach about 20% (Gaurav et al., 2021).

## 5 Summary and outlook

Cell derived vesicles have a natural phospholipid bilayer structure and enclosed compartments, making them a natural drug delivery carrier. At present, various cell-derived vesicles, including tumor cells and immune cells, could be used as drug delivery carriers. Cell sources can be selected according to drug delivery needs. To improve the efficacy of chemotherapy drugs and reduce the toxic side effects, tumor cell-derived vesicles with homing effect can be selected. To improve the immune activation effect, immune cell-derived vesicles with immune regulatory function can be selected. In addition, hybrid vesicles based on the fusion with two or more cell-derived vesicles or the fusion of cell-derived vesicles with liposomes have been developed to address the limitations of the biological function and drug loading capacity of cell-derived vesicles. The mainstream drug loading methods currently include co incubation, extrusion, ultrasound, electroporation, and transfection based on the physicochemical properties of the loaded drugs and the biological structural characteristics of the vesicles. Small molecule drugs can be loaded into vesicles through co incubation, ultrasound, or electroporation, while the preferred loading method for nucleic acid drugs is electroporation.

Compared to traditional liposomes, cell derived vesicles carry a wider range of drugs. With their natural lipid bilayer, cell-derived vesicles have excellent natural targeting properties, which can avoid drug degradation and efficiently deliver drugs to target cells to exert their effects (Kim et al., 2024). In terms of production cost, the production of cell derived vesicles usually requires starting from cell culture, involving steps such as cell culture, vesicle isolation, and purification. Compared with traditional carriers, and its production process is relatively complex and the cost is higher. To obtain high purity and cell derived vesicles that meets quality standards, strict purification and quality control are required, which further increases production costs (Ng et al., 2022). The production of cell derived vesicles is limited by cell sources and yield, and faces certain challenges in large-scale production. However, the possibility of personalized modification of cell-derived vesicles is higher (Pham et al., 2021).

The research on cell-derived vesicles as drug delivery carriers for tumor therapy is gradually becoming standardized. For example, tumor cell-derived vesicles should eliminate the potential carcinogenic risk caused by residual genetic material, and immune cell-derived vesicles should consider whether the intensity of the immune response they trigger is within a safe range. In addition, for further promoting the application of cell derived vesicles in clinical cancer treatment, further research should be focused on the stability of vesicle expansion production, storage, and transportation problems, like limited production capacity, low extraction and purification efficiency, lack of stable production system, poor storage stability, low component stability, and insufficient functional stability.
